# Bilirubin as a Modulator of WNK1 Protein Signaling: Implications for Neuroinflammatory Diseases

**DOI:** 10.1002/advs.202520531

**Published:** 2026-03-20

**Authors:** Sri Jayanti, Libor Vítek, Silvia Gazzin, Claudio Tiribelli

**Affiliations:** ^1^ Eijkman Research Center for Molecular Biology National Research and Innovation Agency of Indonesia Jakarta Indonesia; ^2^ General University Hospital and 1st Faculty of Medicine Charles University Prague Czech Republic; ^3^ Liver‐Brain Unit “Rita Moretti,” Fondazione Italiana Fegato‐ONLUS Trieste Italy

**Keywords:** bilirubin, neurodegenerative diseases, neuroinflammation, neuroprotection, WNK1

## Abstract

Previously regarded merely as a potentially harmful waste product of heme catabolism, bilirubin has now emerged as a pleiotropic molecule with potent antioxidant, anti‐inflammatory, and hormone‐like properties. Recent findings have revealed protective effects against cardiovascular, metabolic, autoimmune, and neoplastic diseases, as well as neurological disorders. The growing understanding of neuroinflammatory processes has opened new avenues for exploring the role of bilirubin in neuroprotection. Despite the increasing number of studies investigating the protective effects of bilirubin in neurological diseases, the molecular mechanisms underlying its anti‐inflammatory actions in the brain remain insufficiently understood. In this perspective article, With‐No‐Lysine (K) kinase 1 (WNK1) is discussed as a newly identified molecular target of bilirubin, highlighting its potential implications for neuroinflammatory diseases. The bilirubin–WNK1 axis may represent a previously unrecognized protective mechanism that could be leveraged to mitigate inflammation‐driven neurodegeneration. This interaction can open new therapeutic opportunities for neurodegenerative disorders such as multiple sclerosis (MS), Alzheimer's disease, and Parkinson's disease.

## 1 A Paradigm Shift: Bilirubin as Both Foe and Friend

Like Dr. Jekyll and Mr. Hyde, bilirubin has a dual nature. At elevated concentrations (hyperbilirubinemia), particularly in newborns, it can be harmful, leading to bilirubin‐induced neurotoxicity that can cause death or manifest as kernicterus spectrum disorder (KSD), a condition arising from region‐specific brain injury [1]. Beyond its well‐demonstrated role in neonatal neurotoxicity, including neuroinflammation, glutamate toxicity, and oxidative stress, high levels of bilirubin can also potentiate the non‐selective cation channels, such as acid‐sensing ion channels (ASICs) [2] and transient receptor potential melastatin 2 (TRPM2) channels [3]. This potentiation increases intracellular Ca^2+^ concentrations and promotes neuronal death.

**FIGURE 1:**
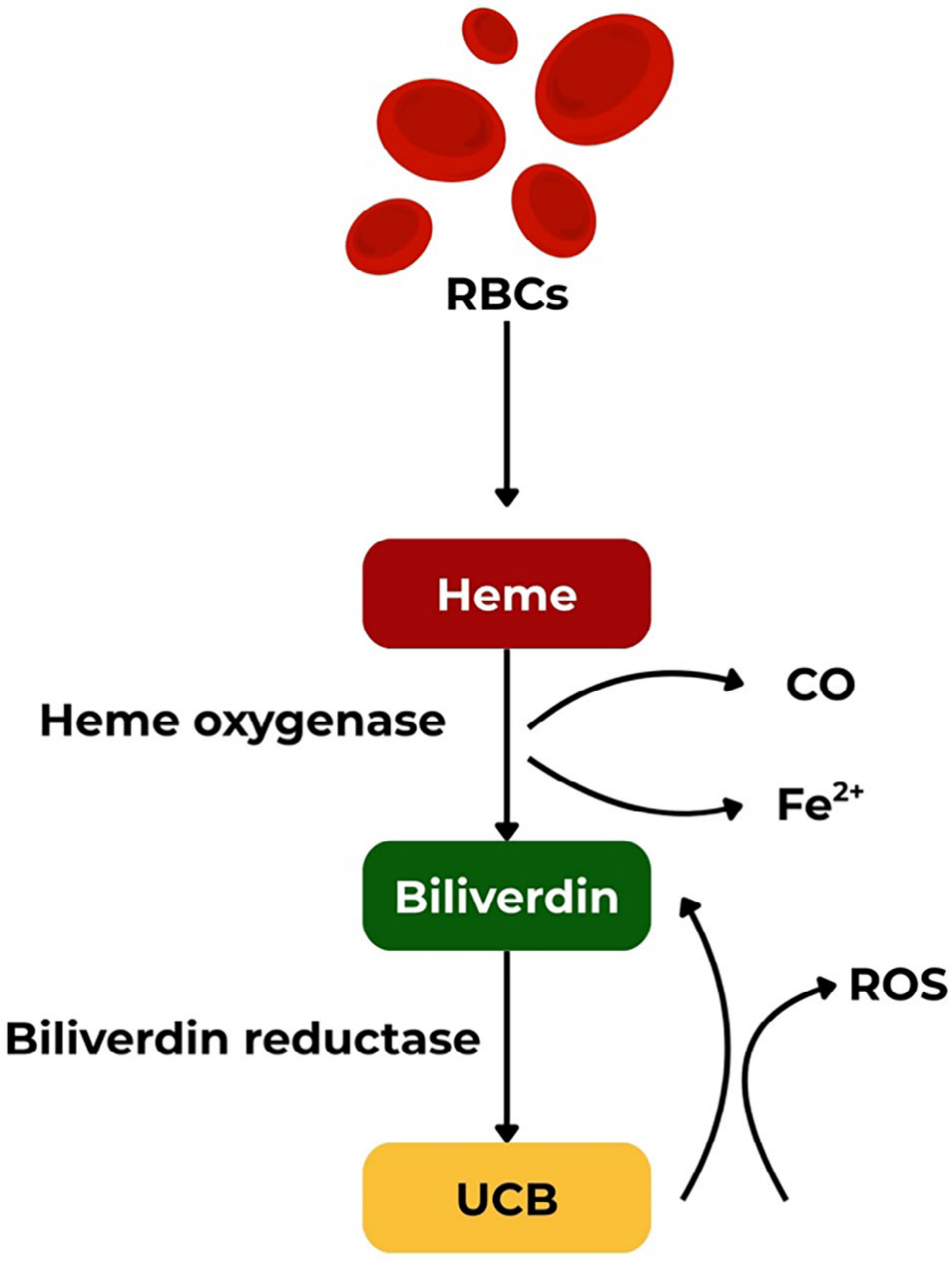
Unconjugated bilirubin (UCB) is generated from the breakdown of heme in red blood cells (RBCs). Heme oxygenase catalyzes its conversion to biliverdin, carbon monoxide (CO), and iron (Fe^2^^+^), after which biliverdin is reduced to UCB by biliverdin reductase. The remarkable antioxidant capacity of bilirubin has been attributed to the bilirubin–biliverdin redox cycle, in which bilirubin is oxidized by reactive oxygen species (ROS) back to biliverdin, which is then rapidly reduced again by biliverdin reductase to regenerate bilirubin.

In contrast, at physiological and slightly supraphysiological concentrations, typically above 10 µmol/L (0.6 mg/dL), bilirubin confers multiple health benefits [4]. Despite this, bilirubin has long been viewed as simply a waste product: the final metabolite of heme catabolism in the vascular bed, destined for excretion. This raises an intriguing question: Why would the body invest so much energy in a two‐step enzymatic process – via heme oxygenases and biliverdin reductases (Figure 1) – simply to generate a pigment long regarded as biologically useless? The first pieces of evidence appeared as early as 50's of the last century, but the right answer began to emerge in 1987, when the first scientific evidence demonstrated that bilirubin is a powerful endogenous antioxidant [5]. Since then, physiological concentrations of bilirubin have been increasingly linked to protective functions throughout life: from neuroprotection in neonatal hypoxic‐ischemic encephalopathy to reducing the risk of postoperative delirium in geriatric patients [6, 7].

## 2 Beyond the Antioxidant Paradigm of Bilirubin

Mildly elevated bilirubin concentrations, such as those observed in individuals with Gilbert syndrome, have been associated with protection against diseases driven by increased oxidative stress, including cardiovascular and metabolic disorders, diabetes, autoimmunity, and cancer [8, 9]. Initially, these protective associations reinforced the prevailing view of bilirubin as primarily an antioxidant. However, a broader perspective has since emerged.

In fact, bilirubin has been rediscovered as a homeostatic regulator with antioxidant, anti‐inflammatory, and even hormone‐like properties [4, 8, 9]. Its pleiotropic actions include modulation of cell signaling, metabolism, and immune responses, with tangible clinical and therapeutic implications. These diverse effects are mediated through the interaction of bilirubin with nuclear and membrane receptors, including those that govern energy homeostasis (peroxisome proliferator‐activated receptors, PPARs, aryl hydrocarbon receptor, AhR; constitutive androstane receptor, CAR), biotransformation pathways (CAR; pregnane X receptor, PXR), and sensory perception (MRGPRX4). In addition, bilirubin can bind with high affinity to metabolic regulators such as fatty acid‐binding protein 1 (FABP1) and apolipoprotein D (apoD), thus modulating further downstream signaling cascades [4]. Notably, bilirubin also exerts powerful effects on immune functions. It suppresses the activity of antigen‐presenting cells and T‐cells, inhibits adhesion molecule expression, and limits the migration of immune cells [9].

Taken together, these insights underscore that bilirubin is neither a merely terminal byproduct of heme catabolism nor solely an antioxidant. Instead, it acts as a multifaceted signaling and regulatory molecule, capable of orchestrating diverse cellular and systemic responses. Recognizing bilirubin as a genuine endocrine mediator reframes its physiological and therapeutic potential, opening new avenues for interventions that harness its broad‐spectrum protective effects.

## 3 Bilirubin and Its Protective Role in Neuroinflammation

**FIGURE 2:**
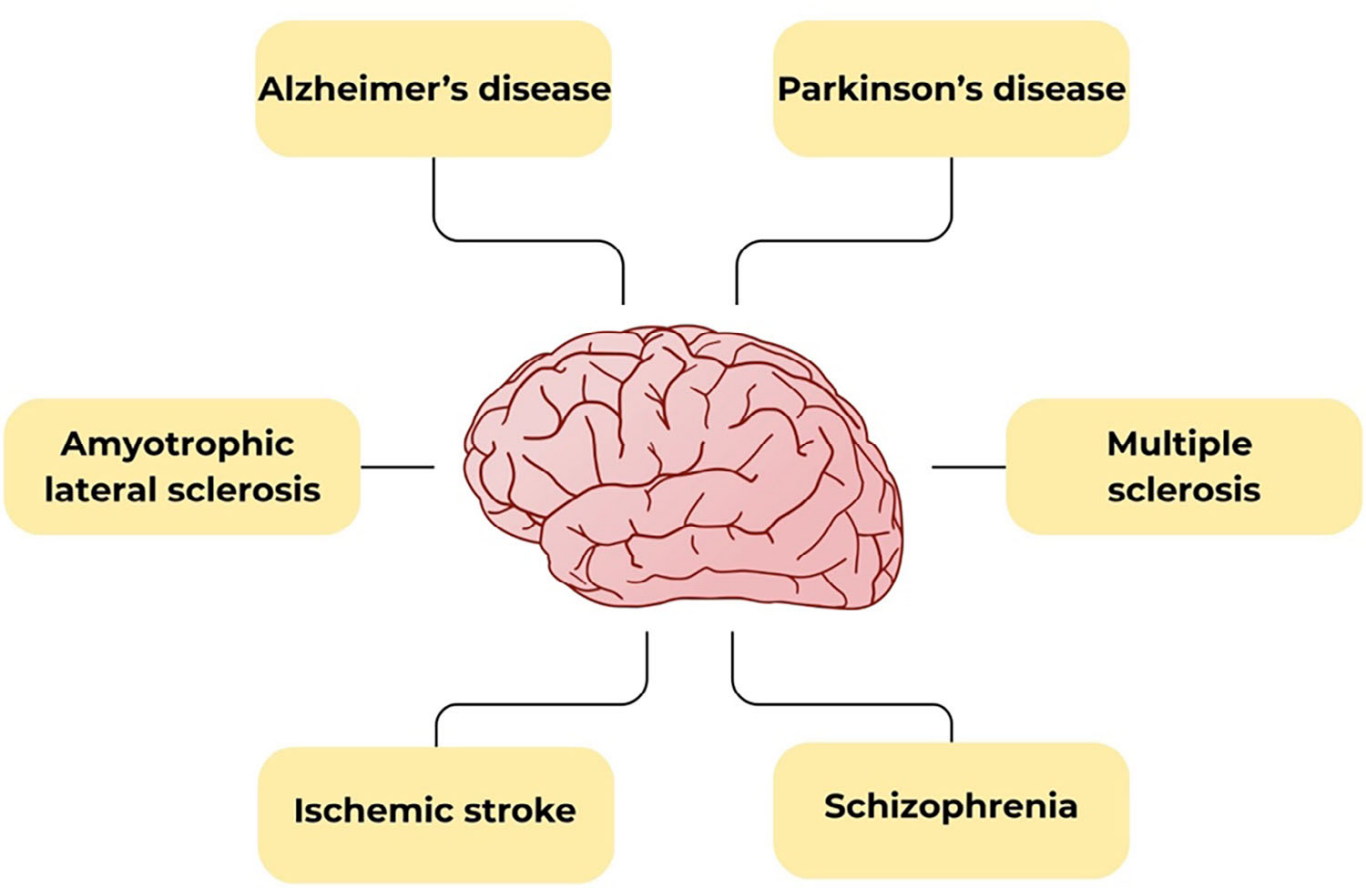
Neurological conditions associated with lower bilirubin concentrations.

Similar to observations in cancer and metabolic disorders, the protective effects of bilirubin have also been observed in neurological diseases. Clinical observations consistently reported substantially lower serum bilirubin concentrations (hypobilirubinemia) in patients with Alzheimer's disease, amyotrophic lateral sclerosis, multiple sclerosis (MS), Parkinson's disease, ischemic stroke, and schizophrenia (Figure 2) [10]. These associations suggest that reduced bilirubin concentrations may reflect loss of its endogenous protection in neurological diseases and/or high consumption during high oxidative stress accompanying neuroinflammatory diseases. This has been convincingly documented in our very recent large clinical study in almost 2,700 patients with MS followed for more than seven years: not only did they have significantly lower serum bilirubin concentrations, but their serum bilirubin was negatively correlated with disability status as well as brain volume [11].

In fact, low serum bilirubin concentration has been shown to disrupt brain regulatory networks of protein phosphorylation, triggering neuroinflammation, promoting neuronal apoptosis, and impairing neuronal growth [12]. By using Blvra^−/−^ mice (a genetically engineered model lacking biliverdin reductase A (Blvra) and thus reducing the generation of bilirubin), proteomic analysis revealed upregulation of the phosphoinositide 3‐kinase/protein kinase B (PI3K‐Akt) signaling pathway, while phosphoproteomic profiling demonstrated increased markers of neuronal apoptosis [12]. These molecular alterations were corroborated by histological findings of more extensive brain lesions, supporting the notion that hypobilirubinemia can drive neurodegeneration. The PI3K‐Akt pathway is well recognized for its role in promoting cell growth, proliferation, and survival, and its modulation has been implicated in mitigating inflammation‐related injury and oxidative stress. In addition, hypobilirubinemia was associated with increased expression of protein kinase C delta (PRKCD) and mitogen‐activated protein kinase 8 (MAPK8), which are critically involved in regulating neuronal apoptosis [12]. Taken together, these findings highlight bilirubin as a potential neuroprotective regulator, whose deficiency can exacerbate vulnerability to neuroinflammatory and neurodegenerative processes.

While most preclinical studies have emphasized the antioxidant properties of bilirubin, growing evidence indicates that its neuroprotective effects extend far beyond the regulation of oxidative stress. In particular, bilirubin has emerged as a potent anti‐inflammatory and immunomodulatory agent in the context of neuroinflammation (Table 1). Its ability to modulate inflammatory processes is increasingly recognized as a central mechanism of protection.

**TABLE 1:** Bilirubin‐mediated mechanisms and targets in neuroinflammation.

Target	Effect	Outcome	Refs.
NF‐κB	Prevents NF‐κB nuclear translocation	Reduces pro‐inflammatory cytokines (IL‐6, TNF‐α, IL‐1β)	[13, 14]
NLRP3 inflammasome	Inhibits NLRP3 activation and IL‐1β release	Limits excessive inflammasome‐mediated neuroinflammation	[15, 16]
TNF‐α	Reduces TNF‐α production	Prevents dopaminergic neuron loss	[17]
CD4+ T cell, MHC class II	Suppresses CD4+ T cell reactivity, reduces MHC class II expression, and downregulates costimulatory molecule	Restrains peripheral and CNS immune activation	[13]
WNK1‐SPARK/OSR1	Bilirubin binds WNK1→ activates SPAK/OSR → increases Cl^−^ homeostasis	Dampens NLRP3 inflammasome activity and reduces neuroinflammasome	[16]

CNS, central nervous system; IL, interleukin; MHC, major histocompatibility complex; NF‐κB, nuclear factor‐kappa B; NLRP3, NOD‐like receptor family pyrin domain‐containing 3; TNF‐α, tumor necrosis factor alpha; WNK1‐SPAK/OSR1, with‐no‐lysine (K) protein kinase 1‐STE20/SPS1‐related proline‐ and alanine‐rich kinase/oxidative stress‐responsive 1 kinase.

**FIGURE 3:**
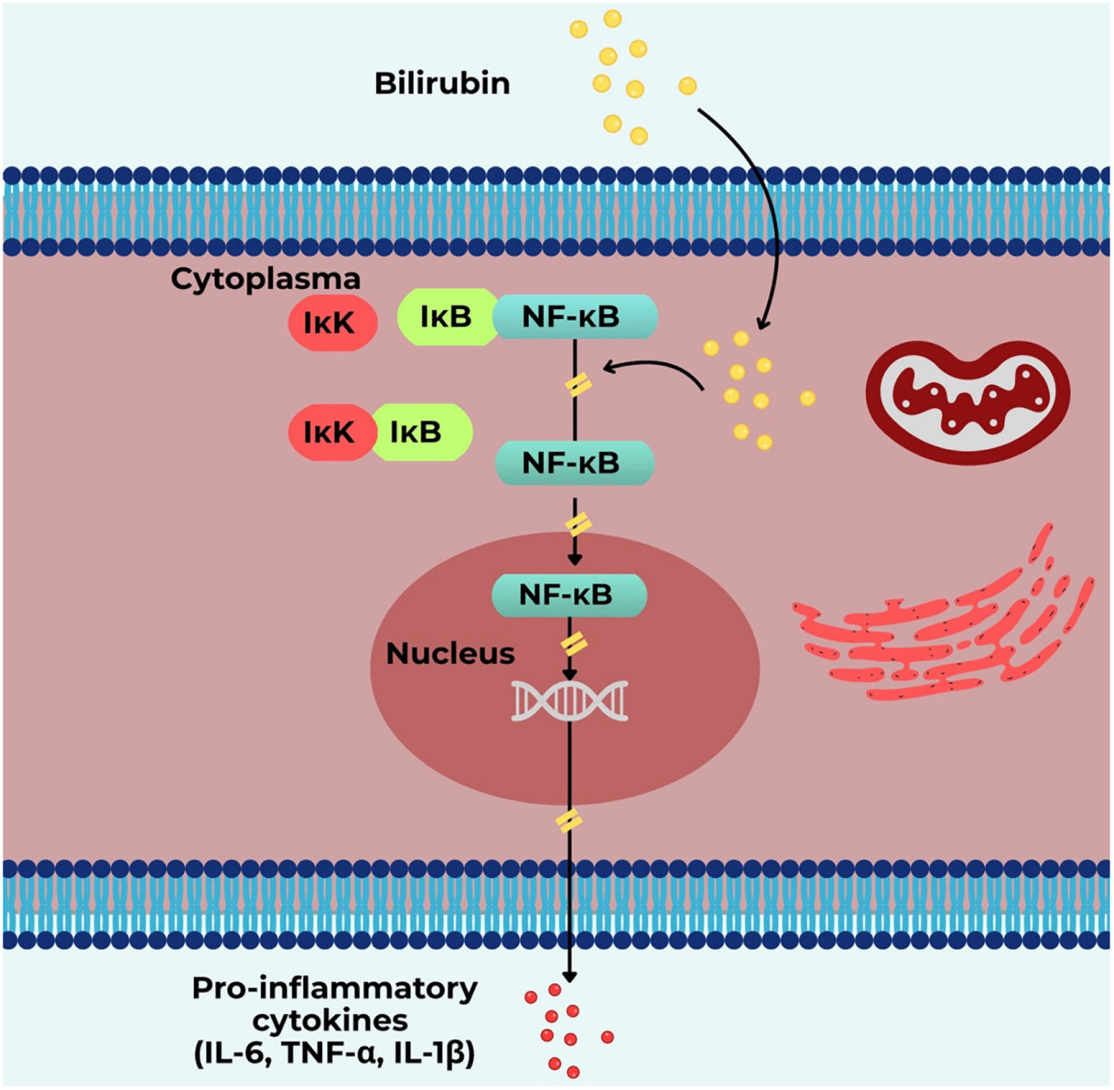
Bilirubin exerts its anti‐inflammatory effect by interfering with the NF‐κB signaling cascade. Physiologically, NF‐κB is retained in the cytoplasm by the inhibitor of the nuclear factor‐kappa B (IκB), but upon inflammatory stimulation, IκB kinase (IKK) phosphorylates and degrades IκB, releasing NF‐κB to translocate to the nucleus and promote transcription of pro‐inflammatory mediators such as interleukin‐6 (IL‐6), tumor necrosis factor‐alpha (TNF‐α), and IL‐1β. By blocking this process, bilirubin suppresses NF‐κB activation and downstream inflammatory responses.

One of the best‐characterized pathways involves the regulation by bilirubin of nuclear factor‐kappa B (NF‐κB), a master regulator of inflammation (Figure 3) [13]. Under physiological conditions, NF‐κB remains inactive in the cytoplasm by binding to the inhibitor of nuclear factor‐kappa B (IκB). Upon inflammatory stimulation, IκB kinase phosphorylates and degrades IκB, allowing NF‐κB to translocate into the nucleus and initiate the transcription of pro‐inflammatory mediators [14]. In experimental autoimmune encephalomyelitis, a model of MS, bilirubin interferes with this cascade, thus limiting NF‐κB‐driven gene expression [13].

Beyond NF‐κB, bilirubin also modulates adaptive immune responses by suppressing CD4+ T cell reactivity, reducing the expression of MHC class II, and downregulating costimulatory molecules in both T cells and macrophages [13]. These immunomodulatory effects underscore the broader role of bilirubin in dampening neuroinflammatory signaling and maintaining neural homeostasis.

Bilirubin also inhibits another key inflammatory pathway, the inflammasome. At physiological concentrations, bilirubin attenuates the activation of the NLRP3 (NOD‐like receptor family pyrin domain‐containing 3) inflammasome, a cytosolic multiprotein complex that senses cellular stress and promotes IL‐1β production [15]. Excessive activation of the inflammasome is detrimental to the host and has been strongly implicated in the pathogenesis of neurodegenerative diseases, including Alzheimer's and Parkinson's diseases [18].

In addition, bilirubin reduces the production of tumor necrosis factor alpha (TNF‐α), a potent pro‐inflammatory cytokine [15, 17]. In the model of Parkinson's disease, the effect of bilirubin on TNF‐α was found to be as effective as treatment with an anti‐TNF‐α drug. Importantly, the ability of bilirubin to prevent loss of dopaminergic neurons in the substantia nigra (the neuropathological hallmark underlying motor deficits in Parkinson's disease) was not replicated by the potent antioxidant, N‐acetylcysteine [10]. This observation suggests that neuroinflammation, rather than oxidative stress, is the main driver of the pathogenesis of Parkinson's disease, and that bilirubin confers protection predominantly through its anti‐inflammatory activity.

## 4 WNK1: A Novel Bilirubin Target in Neuroinflammation

While the anti‐inflammatory capacities of bilirubin have increasingly gained attention, its precise molecular targets and downstream signaling mechanisms remain only partially understood. A recent discovery by Mao et al. adds an intriguing new dimension by identifying with‐no‐lysine (K) kinase 1 (WNK‐1) as a direct binding target of bilirubin in the context of neuroinflammation [16]. WNK1, a serine‐threonine kinase, is a central component of signaling networks that regulate chloride ion homeostasis, immune regulation, autophagy, and other essential cellular processes.

**FIGURE 4:**
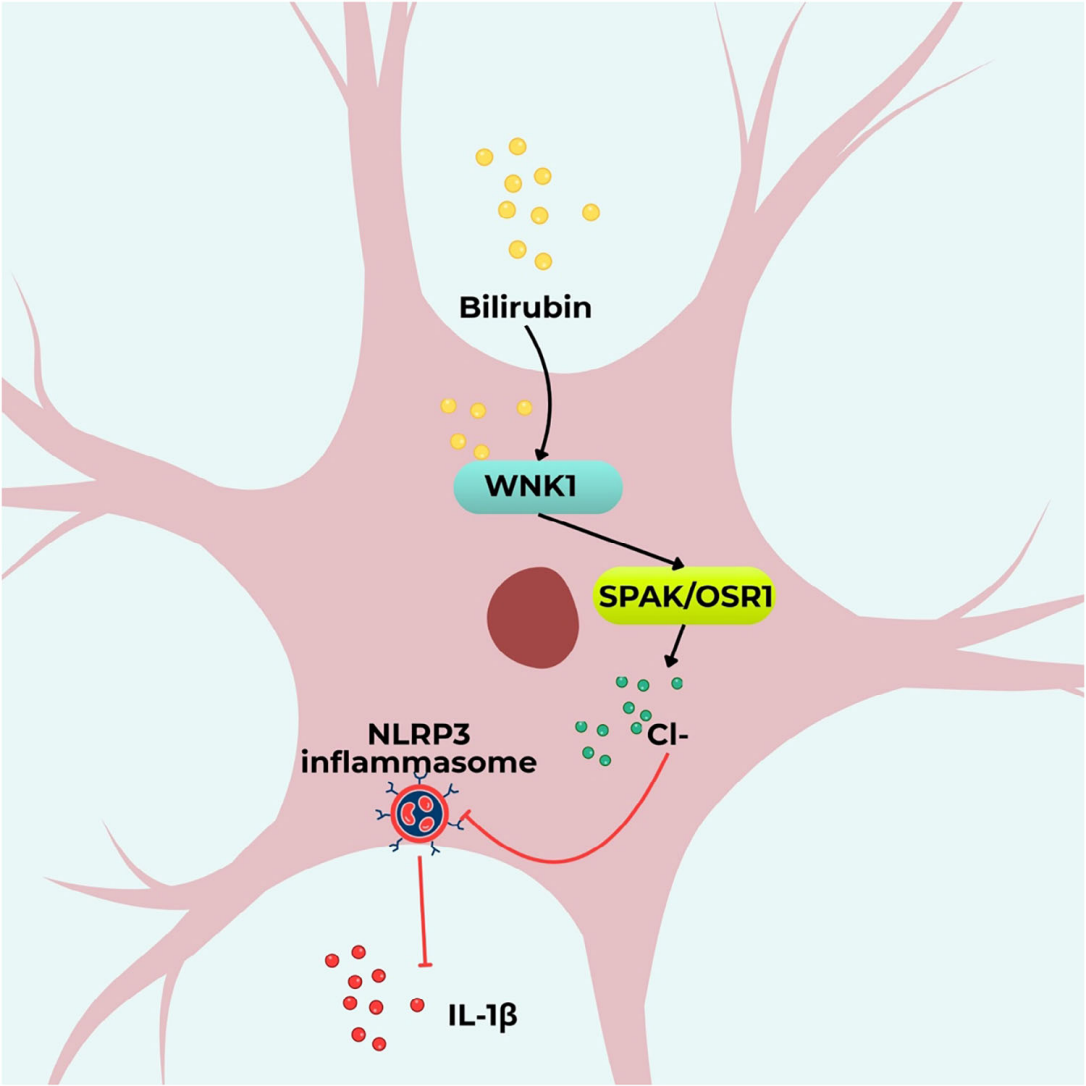
The bilirubin–WNK1 interaction suppresses NLRP3 inflammasome activation as a novel anti‐inflammatory mechanism. Bilirubin directly interacts with WNK1, enhancing its kinase activity and promoting the activation of its downstream targets, Ste20‐related proline alanine‐rich kinase (SPAK) and oxidative stress‐responsive kinase 1 (OSR1) in neurons. This signaling cascade increases intracellular chloride ion levels, which in turn suppresses NOD‐like receptor family pyrin domain‐containing 3 (NLRP3) inflammasome activation and attenuates neuroinflammation.

Bilirubin interacts directly with WNK‐1, enhancing its kinase activity and activating its downstream target Ste20‐related proline alanine‐rich kinase (SPAK) and oxidative stress‐responsive kinase 1 (OSR1) in neurons. This signaling cascade increases intracellular chloride ion concentrations, thus suppressing NLRP3‐mediated neuroinflammation. Notably, the binding affinity of bilirubin for WNK‐1 occurs at concentrations comparable to its physiological levels in vivo, underscoring the translational significance of this interaction [16]. These findings highlight WNK‐1 as a negative regulator of the NLRP‐3 inflammasome and position the direct binding of bilirubin to WNK‐1, likely through conformational modulation and/or enhancement of kinase activity, as a novel mechanism underlying its anti‐inflammatory effects (Figure 4).

Moreover, WNK1 also functions as an immunomodulator. Its knockdown promotes the nuclear translocation of NF‐κB and activates the p38 and Jun N‐terminal kinase (JNK) mitogen‐activated protein kinase (MAPK) signaling pathway in macrophages, leading to proinflammatory cytokine production, including IL‐6 and TNF‐α [19]. This suggests a potential synergistic effect with bilirubin, which is known to target NF‐κB signaling [13, 14].

Beyond its kinase domain, WNK‐1 contains a large intrinsically disordered C‐terminal domain that drives phase separation, enabling it to sense molecular crowding and trigger ion transport responses that restore cell volume [20]. Molecular crowding has been implicated in protein aggregation processes in neurodegenerative diseases, such as those involving α‐synuclein, amyloid‐β, and tau protein. Depending on the specific crowding agents, molecular crowding may either promote or inhibit aggregation [21].

Although there is currently no direct evidence that bilirubin binds to the C‐terminal domain of WNK1, this possibly opens an avenue for future investigation. Given the potent antioxidant capacity of bilirubin and its ability to protect cellular membranes by scavenging lipophilic radicals [22], such interactions could contribute to the preservation of cell integrity and potentially limit the accumulation of intracellular effects of molecular crowding‐associated factors.

Mao et al. discovery repositions WNK1 in the broader landscape of neurological diseases. Although WNK1 has not been extensively studied as a bilirubin target or a neuroinflammatory regulator, its abundant expression in the cerebral cortex and cerebellum, and RNA enrichment in oligodendrocytes [23] suggest that its relevance in brain physiology may be greater than previously recognized

Most prior work in the field of neurological diseases has focused on the role of WNK1 in hereditary sensory and autonomic neuropathy type II (HSANII). Mutations in a neural‐specific splice variant of WNK1 (HSN2) cause HSANII, an autosomal neuropathy characterized by sensory dysfunction (loss of pain, touch, and temperature sensation and limb abnormalities [e.g, finger deformities and Charcot's joint]). Mechanistic studies highlighted dysregulated neurite outgrowth mediated by OSR1 activation and glycogen synthase kinase 3β (GSK3β) signaling [24]. Beyond HSANII, WNK‐1 has also been implicated in ischemic stroke, where its hyperactivation enhances NF‐κB transcriptional activity, aggravating the development of brain lesions and worsening neurological deficits [25, 26]. Additionally, WNK1 is being explored as a therapeutic target in glioblastoma, where reduced WNK1 catalytic activity disrupts intracellular ion homeostasis, leading to cancer cell death [27].

Taken together, these findings suggest a dual role of WNK‐1 in neurological diseases: in some contexts, its activation exerts protective effects, whereas in others, its inhibition proves beneficial. This functional duality highlights the importance of context‐specific strategies when considering WNK1 as a therapeutic target. The discovery that bilirubin directly modulates WNK1 gives an additional layer of complexity, raising the possibility that the beneficial effects of bilirubin in neurodegenerative diseases may, at least in part, be mediated through WNK1‐dependent signaling pathways.

From a broader perspective, recent findings of Mao et al. identify WNK1 not only as a novel target of bilirubin [16] but also as a potentially underappreciated regulator of neuroinflammation. More studies are needed to determine how the interactions between bilirubin and WNK1 differ across brain cell types, how they intersect with oxidase stress and autophagy pathways, and whether they can be used to selectively regulate the activity of NLRP3 in neurodegenerative diseases such as Alzheimer's disease, Parkinson's disease, and MS.

## 5 Conclusion

Bilirubin is an intriguing molecule, and the study by Mao and colleagues adds yet another piece to this increasingly complex puzzle. Its protective effects against neuroinflammation may prove crucial in addressing the rapidly growing burden of neurological diseases in which inflammation plays a central role, including Parkinson's and Alzheimer's disease. The future will reveal whether what was once considered merely a “waste product of heme” might instead hold therapeutic potential to mitigate the effects of brain aging and neurodegeneration.

## Funding

This work was supported by an internal grant from the National Research and Innovation Agency of Indonesia to S.J; by grant of MH CZ‐DRO‐VFN64165 from the Czech Ministry of Health and the Inter‐Action project LUAUS26259, given by the Czech Ministry of Education to L.V., and by an internal grant from Fondazione Italiana Fegato‐ONLUS to S.G. and C.T.

## Conflicts of Interest

The authors declare no conflicts of interest.

## References

[1] S. Gazzin , F. Masutti , L. Vítek , and C. Tiribelli , “The Molecular Basis of Jaundice: An Old Symptom Revisited,” Liver International 37 (2016): 1094–1102, 10.1111/liv.13351. 28004508

[2] K. Lai , X.‐L. Song , H.‐S. Shi , et al., “Bilirubin Enhances the Activity of ASIC Channels to Exacerbate Neurotoxicity in Neonatal Hyperbilirubinemia in Mice,” Science Translational Medicine 12, no. 530 (2020): aax1337, 10.1126/scitranslmed.aax1337. 32051225

[3] H.‐W. Liu , L.‐N. Gong , K. Lai , et al., “Bilirubin Gates the TRPM2 Channel as a Direct Agonist to Exacerbate Ischemic Brain Damage,” Neuron 111, no. 10 (2023): 1609–1625.e6, 10.1016/j.neuron.2023.02.022. 36921602 PMC10191619

[4] L. Vítek and C. Tiribelli , “Bilirubin: The Yellow Hormone?” Journal of Hepatology 75, no. 6 (2021): 1485–1490, 10.1016/j.jhep.2021.06.010. 34153399

[5] R. Stocker , Y. Yamamoto , A. F. McDonagh , A. N. Glazer , and B. N. Ames , “Bilirubin is an Antioxidant of Possible Physiological Importance,” Science 235, no. 4792 (1987): 1043–1046, 10.1126/science.3029864. 3029864

[6] R. Yu , M. Yang , J. Chen , and F. Zhang , “The Relationship Between Preoperative Serum Indirect Bilirubin and Postoperative Delirium in Geriatric Patients Undergoing Joint Replacement,” PLoS ONE 20, no. 3 (2025): 0320719, 10.1371/journal.pone.0320719. PMC11949354 40146749

[7] L. Zou , H. Yuan , Q. Liu , C. Lu , and L. Wang , “Potential Protective Effects of Bilirubin Following the Treatment of Neonatal Hypoxic‐ischemic Encephalopathy with Hypothermia Therapy,” Bioscience Reports 39, no. 6 (2019): BSR20182332, 10.1042/BSR20182332. 31101685 PMC6549084

[8] L. Vitek , “The Role of Bilirubin in Diabetes, Metabolic Syndrome, and Cardiovascular Diseases,” Frontiers in Pharmacology 3 (2012): 55, 10.3389/fphar.2012.00055. 22493581 PMC3318228

[9] S. Gazzin , L. Vitek , J. Watchko , S. M. Shapiro , and C. Tiribelli , “A Novel Perspective on the Biology of Bilirubin in Health and Disease,” Trends in Molecular Medicine 22, no. 9 (2016): 758–768, 10.1016/j.molmed.2016.07.004. 27515064

[10] S. Jayanti , R. Moretti , C. Tiribelli , and S. Gazzin , “Bilirubin and Inflammation in Neurodegenerative and Other Neurological Diseases,” Neurosciences 7, no. 2 (2020): 92–108, 10.20517/2347-8659.2019.14.

[11] T. Uher , P. Kleinová , J. Woronyczová , et al., “Serum Bilirubin Concentrations and Their Association With Clinical and Radiological Outcomes in Multiple Sclerosis: A Large Cohort Study,” Annals of Hepatology 31, no. 1 (2025): 102117, 10.1016/j.aohep.2025.102117. 40992627

[12] M. Chen , C. Wu , Y. Cui , et al., “Phosphoproteomics Profiling Reveals Key Proteins Involved in Neuroinflammation and Impaired Axon Guidance Induced by Bilirubin Deficiency,” ACS Chemical Neuroscience 16, no. 14 (2025): 2602–2616, 10.1021/acschemneuro.5c00117. 40621869

[13] Y. Liu , P. Li , J. Lu , et al., “Bilirubin Possesses Powerful Immunomodulatory Activity and Suppresses Experimental Autoimmune Encephalomyelitis,” The Journal of Immunology 181, no. 3 (2008): 1887–1897, 10.4049/jimmunol.181.3.1887. 18641326

[14] Y. Zhang , H. Luan , and P. Song , “Bilirubin Metabolism and its Application in Disease Prevention: Mechanisms and Research Advances,” Inflammation Research 74, no. 1 (2025): 81, 10.1007/s00011-025-02049-w. 40413269

[15] Y. Li , H. Sheng , Z. Yan , et al., “Bilirubin Stabilizes the Mitochondrial Membranes During NLRP3 Inflammasome Activation,” Biochemical Pharmacology 203 (2022): 115204, 10.1016/j.bcp.2022.115204. 35944727

[16] L. Mao , J. Lu , Q. Yang , et al., “Bilirubin Targeting WNK1 to Alleviate NLRP3‐Mediated Neuroinflammation,” Advanced Science 12, no. 29 (2025): 2407349, 10.1002/advs.202407349. 40112213 PMC12362796

[17] S. Jayanti , R. Moretti , C. Tiribelli , and S. Gazzin , “Bilirubin Prevents the TH^+^ Dopaminergic Neuron Loss in a Parkinson's Disease Model by Acting on TNF‐α,” International Journal of Molecular Sciences 23, no. 22 (2022): 14276, 10.3390/ijms232214276. 36430754 PMC9693357

[18] H. Guo , J. B. Callaway , and J. P.‐Y. Ting , “Inflammasomes: Mechanism of Action, Role in Disease, and Therapeutics,” Nature Medicine 21, no. 7 (2015): 677–687, 10.1038/nm.3893. PMC4519035 26121197

[19] Y. Arai , K. Asano , and S. Mandai , et al., “WNK1–TAK1 Signaling Suppresses Lipopolysaccharide‐Induced Cytokine Production and Classical Activation in Macrophages,” Biochemical and Biophysical Research Communications 533, no. 4 (2020): 1290–1297, 10.1016/j.bbrc.2020.10.007. 33046244

[20] C. R. Boyd‐Shiwarski , D. J. Shiwarski , S. E. Griffiths , et al., “WNK Kinases Sense Molecular Crowding and Rescue Cell Volume via Phase Separation,” Cell 185, no. 24 (2022): 4488–4506.e20, 10.1016/j.cell.2022.09.042. 36318922 PMC9699283

[21] S. P. Patel , T. Nikam , B. Sreepathi , et al., “Unraveling the Molecular Jam: How Crowding Shapes Protein Aggregation in Neurodegenerative Disorders,” ACS Chemical Biology 19, no. 10 (2024): 2118–2130, 10.1021/acschembio.4c00365. 39373539

[22] S. Jayanti , R. Moretti , C. Tiribelli , and S. Gazzin , “Bilirubin: A Promising Therapy for Parkinson's Disease,” International Journal of Molecular Sciences 22, no. 12 (2021): 6223, 10.3390/ijms22126223. 34207581 PMC8228391

[23] “Tissue Expression of WNK1 – Summary—The Human Protein Atlas,”accessed September, 01, 2025, https://www.proteinatlas.org/ENSG00000060237‐WNK1/tissue.

[24] M. Shimizu and H. Shibuya , “WNK1/HSN2 Mediates Neurite Outgrowth and Differentiation via a OSR1/GSK3β‐LHX8 Pathway,” Scientific Reports 12, no. 1 (2022): 15858, 10.1038/s41598-022-20271-y. 36151370 PMC9508073

[25] M. I. H. Bhuiyan , C. B. Young , I. Jahan , et al., “NF‐κB Signaling‐Mediated Activation of WNK‐SPAK‐NKCC1 Cascade in Worsened Stroke Outcomes of Ang II–Hypertensive Mice,” Stroke 53, no. 5 (2022): 1720–1734, 10.1161/STROKEAHA.121.038351. 35272484 PMC9038703

[26] M. I. H. Bhuiyan , S. Song , H. Yuan , et al., “WNK‐Cab39‐NKCC1 Signaling Increases the Susceptibility to Ischemic Brain Damage in Hypertensive Rats,” Journal of Cerebral Blood Flow & Metabolism 37, no. 8 (2017): 2780–2794, 10.1177/0271678x16675368. 27798271 PMC5536788

[27] W. Chen , L. N. Zebaze , J. Dong , et al., “WNK1 kinase and Its Partners Akt, SGK1 and NBC‐family Na^+^/HCO3^−^ Cotransporters Are Potential Therapeutic Targets for Glioblastoma Stem‐Like Cells Linked to Bisacodyl Signaling,” Oncotarget 9, no. 43 (2018): 27197–27219, 10.18632/oncotarget.25509. 29930759 PMC6007472

